# Peptidoglycan-Mediated Bone Marrow Autonomic Neuropathy Impairs Hematopoietic Stem/Progenitor Cells via a NOD1-Dependent Pathway in db/db Mice

**DOI:** 10.1155/2022/4249843

**Published:** 2022-08-04

**Authors:** Jing Wu, Binghan Zhang, Shengbing Li, Wenwen Chen, Jinning Mao, Ke Li, Dongfang Liu, Yaqian Duan

**Affiliations:** ^1^Department of Endocrinology and Metabolism, The Second Affiliated Hospital of Chongqing Medical University, Chongqing, China; ^2^Department of Hematology/Oncology, Chongqing University Cancer Hospital, Chongqing, China; ^3^Department of Cardiology, The Second Affiliated Hospital of Chongqing Medical University, Chongqing, China

## Abstract

Impairment of bone marrow-derived hematopoietic stem/progenitor cells (HSPCs) contributes to the progression of vascular complications in subjects with diabetes. Very small amounts of bacterial-derived pathogen-associated molecular patterns (PAMPs) establish the bone marrow cell pool. We hypothesize that alteration of the PAMP peptidoglycan (PGN) exacerbates HSPC dysfunction in diabetes. We observed increased PGN infiltration in the bone marrow of diabetic mice. Exogenous administration of PGN selectively reduced the number of long-term repopulating hematopoietic stem cells (LT-HSCs), accompanied by impaired vasoreparative functions in db/db mouse bone marrow. We further revealed that bone marrow denervation contributed to PGN-associated HSPC dysfunction. Inhibition of NOD1 ameliorated PGN-induced bone marrow autonomic neuropathy, which significantly rejuvenated the HSPC pools and functions in vivo. These data reveal for the first time that PGN, as a critical factor on the gut-bone marrow axis, promotes bone marrow denervation and HSPC modulation in the context of diabetes.

## 1. Introduction

Diabetes disrupts all bodily organ systems, including the bone marrow. Diabetes is associated with impairment of the compartmentalization and function of bone marrow-derived hematopoietic stem/progenitor cells (HSPCs) [1, 2]. HSPCs play a crucial role in vascular repair through paracrine mechanisms [3], and impairment of these cells leads to the progression of microvascular complications in subjects with diabetes [1]. Since dysfunction of HSPC populations strongly contributes to diabetes complications involving vessel degeneration, understanding the underlying mechanisms is critical for revascularization purposes.

The roles of the gut microbiome and pathogen-associated molecular patterns (PAMPs) in the pathogenesis of diabetes microvascular complications have garnered interest [4]. Bacteria-derived PAMPs translocate from the gut into the circulation through disrupted gut barriers in subjects with diabetes, thereby causing systemic effects, including chronic low-grade systemic inflammation, dyslipidemia, and insulin resistance [5, 6]. We previously revealed the bacterial composition and functional profile of the diabetes microbiome. We determined that the levels of peptidoglycan (PGN), a type of PAMP, were substantially elevated in plasma samples from both humans and rodents with type 1 and type 2 diabetes and that increased PGN levels potentially promote diabetes microvascular complications [5, 7]. However, how increased circulating PGN levels affect distant organs, especially the bone marrow, that strongly contribute to microangiopathy in diabetes remains largely unknown.

PGN is a major component of the bacterial cell wall that consists of amino acids and glycan chains of *β*-(1,4)-linked N-acetylglucosamine (NAG) and N-acetylmuramic acid (NAM) repeats [8]. Accumulating evidence suggests that very low concentrations of bacterial antigens control the size of the bone marrow cell pool [9]. A recent study showed that PGN promotes granulopoiesis and induces neutrophil production via a Toll-like receptor 2-dependent pathway [10], suggesting its involvement in gut-bone marrow communication. However, little is known about the impact of PGN on bone marrow HSPC populations. Thus, we herein tested the hypothesis that the bacterial PAMP PGN participates in the regulation of the bone marrow-derived HPSC pool and functions. PGN levels were markedly increased in the bone marrow extracellular fluid harvested from diabetic db/db mice, which was associated with imbalance of the bone marrow hematopoietic stem cell pool and HSPC dysfunction. Our data further showed that autonomic neuropathy underlies the PGN-mediated impairment of HSPC quantity and function and that bone marrow denervation relies on the nucleotide-binding oligomerization domain-containing protein 1- (NOD1-) dependent pathway.

## 2. Materials and Methods

### 2.1. Animals

Male diabetic db/db mice are homozygous for the Lepr^db^ mutation, and db/m mice that were heterozygous for Lepr^db^ were used as controls in this study. Four-week-old male db/db mice, their age-matched db/m controls, and B6.SJL (CD45.1) mice were obtained from the Model Animal Research Center of Nanjing University and housed at the Animal Center of Chongqing Medical University (Chongqing, China). The experiments were conducted according to standard animal care protocols that were approved by the Committee on Animal Research of Chongqing Medical University. Experimental mice were randomly divided into different subgroups, and their blood glucose levels and body weights were measured every two weeks. Six-week-old db/db mice were used if their blood glucose levels exceeded 250 mg/dL in at least two measurements. Glycated hemoglobin was assessed on the day prior to euthanasia by the A1CNow+ kit (Bayer HealthCare, Sunnyvale, CA, US). For PGN treatment, 100 *μ*g of *E. coli* PGN (cat#: tlrl-ksspgn, InvivoGen, San Diego, CA, US) was administered four months after the establishment of diabetes in db/db mice and to their age-matched db/m controls via the tail vein every other day for one week (on the 1^st^, 3^rd^, 5^th^, and 7^th^ days). An equal amount of saline was injected into the mice as a control. In a separate experiment, the NOD1 receptor-specific inhibitor ML-130 (50 *μ*g) (cat#: ab142177, Abcam, Cambridge, MA, US) was administered together with the purified PGN to establish the db/db+PGN+ML130 group. An equal amount of vehicle (DMSO/saline) was administered to age-matched control mice. For sympathetic nervous system disruption, the mice were administered the neuron toxin 6-hydroxydopamine 3 times via intraperitoneal injection according to a previously published protocol [11]. The animals were anesthetized and euthanized by isoflurane inhalation, followed by cervical dislocation at the study end point.

### 2.2. Bone Marrow Extracellular Fluid Collection

The left femoral bone was flushed with ice cold phosphate-buffered saline (PBS) (cat#: 10010023, Thermo Fisher Scientific, Waltham, MA, US). Extracellular fluid was collected by centrifugation at 500 g for 10 minutes at 4°C and stored in liquid nitrogen until analysis.

### 2.3. Measurement of Peptidoglycan Levels

Mouse plasma and bone marrow extracellular fluid were collected for PGN measurement. The experiments were performed using a mouse PGN ELISA kit (cat#: MBS263268 MyBoSource Inc., San Diego, CA, US) according to the manufacturer's instructions as previously described [5]. The absorbance at 450 nm was detected by an ELISA microplate reader.

### 2.4. Bone Marrow Transplantation

For a competitive transplant, 1 × 10^3^ sorted LSK cells (CD45.2) from db/m+vehicle, db/db+vehicle, db/m+PGN, and db/db+PGN mouse groups were mixed with 3 × 10^5^ bone marrow cells (CD45.1, B6.SJL competitor) and injected into lethally irradiated F1 progeny of C57Bl6 x B6.SJL breeding mice (CD45.1^+^ CD45.2^+^, recipient). Engraftment efficacy is determined by donor contribution of CD45.2^+^ cells in peripheral blood at 16 weeks after transplantation which were stained with anti-CD45.2 FITC andanti-CD45.1 PE and analyzed using flow cytometry.

### 2.5. Flow Cytometry

Bone marrow cells were harvested from mouse femurs and tibias and washed with PBS. The cells were then treated with 5% rat serum for 15 minutes at 4°C, followed by incubation with the primary antibody for 30 minutes at 4°C in the dark. After rinsing with PBS the next day, the cells were stained with Flexible Viability Dye and fixed with 1% paraformaldehyde (PFA). The following antibody cocktail was used: BV421 anti-mouse lineage cocktail, Biolegend, cat#: 133311; PE-CF594 anti-mouse CD127, BD Biosciences, cat#: 562419; PE/Cy7 anti-mouse Sca-1, Biolegend, cat#: 108114; FITC anti-mouse c-Kit, Biolegend, San Diego, CA, cat#: 105806; PE anti-mouse CD34, Biolegend, cat#: 119; PerCP-eFluor 710 anti-mouse CD135, eBioscience, San Diego, CA, cat#: 46–1351-82; and APC anti-mouse CD16/CD32, eBioscience, cat#: 14–0161-82.

### 2.6. Lineage^−^c-Kit^+^ Cell Isolation and Treatment

Bone marrow lineage^−^c-Kit^+^ (LK) HSPCs were separated by EasySep magnets (cat #: 18000, STEMCELL Technologies, Vancouver, BC, Canada) using lineage-negative and c-Kit-positive selection kits (cat #s: 19856 and 18757, STEMCELL Technologies, Vancouver, BC, Canada). To determine the effects of PGN on HSPCs ex vivo, the cells were pretreated with purified PGN (100 *μ*g/mL), for 2 h in culture medium [12].

### 2.7. Migration Assay

The migratory function of bone marrow LK cells was determined based on their migration towards a chemoattractant CXCL12 gradient using the fluorometric QCM 96-well chemotaxis cell migration assay (ECM512, Millipore, Temecula, CA, US) as previously described [13].

### 2.8. Proliferation Assay

Bone marrow LK cells were cultured in serum-free StemSpan media with or without *E. coli* PGN supplementation, and their proliferation was then assayed with a BrdU ELISA kit (cat#: 11647229001, Roche, Indianapolis, IN, US) according to the manufacturer's instructions. For the BrdU incorporation analysis in vivo, experimental mice were received 3 mg BrdU via intraperitoneal injection once and 1 mg/mL of BrdU (Sigma) added to drinking water for 2 days. The analysis of BrdU incorporation was then performed using the FITC BrdU flow kit (BD Biosciences, cat#: 559619) as previously reported [14].

### 2.9. CFU Assay

Cells from bone marrow or blood were treated with an ammonium chloride solution to lyse the red blood cells and then plated in MethoCult GF M3434 medium (STEMCELL Technologies) according to the manufacturer's instructions as previously described [13].

### 2.10. Long-Term Culture Initiating Cell (LTC-IC) Assay

Primary bone marrow feeder layers were cultured and established using MyeloCult M5300 and then irradiated according to the protocol from StemCell Technologies. 1 × 10^3^ sorted LSK cells were cultured on feeder layers at 33° in 5% CO_2_ for 4 weeks with a weekly half media change. Cells were then harvested and plated into MethoCult GF M3434 in triplicate. Colonies were determined after 12 days and expressed as number of the CFUs per 1 × 10^3^ sorted LSK cells as previously reported [14].

### 2.11. Immunohistochemistry

Paraffin-embedded femoral bones were sectioned and deparaffinized, and the slides were processed as previously described [15]. Briefly, after antigen retrieval and nonspecific protein blockage, the slides were stained with either polyclonal rabbit anti-NF200 (Sigma-Aldrich) or polyclonal rabbit anti-tyrosine hydroxylase (Millipore) at 1 : 100 dilutions. After antibody incubation, the slides were treated with R.T.U. Elite ABC reagent and NovaRED (Vector Laboratories), followed by counterstaining with Gill 2 hematoxylin (Thermo Fisher Scientific).

### 2.12. Data Analysis

All data were evaluated for normal distribution by the JMP 9 software. For multiple comparisons, one-way or two-way ANOVA was used, followed by a post hoc test. The nonparametric Kruskal-Wallis and Mann-Whitney tests were used if the data were not normally distributed. *P* < 0.05 was considered significant. The results are expressed as the mean ± SD.

## 3. Results

### 3.1. Increased Peptidoglycan Levels in the Circulation and Bone Marrow Extracellular Fluid Are Associated with Depletion of the HSPC Pool and Disruption of HSPC Function in db/db Mice

**Figure 1:**
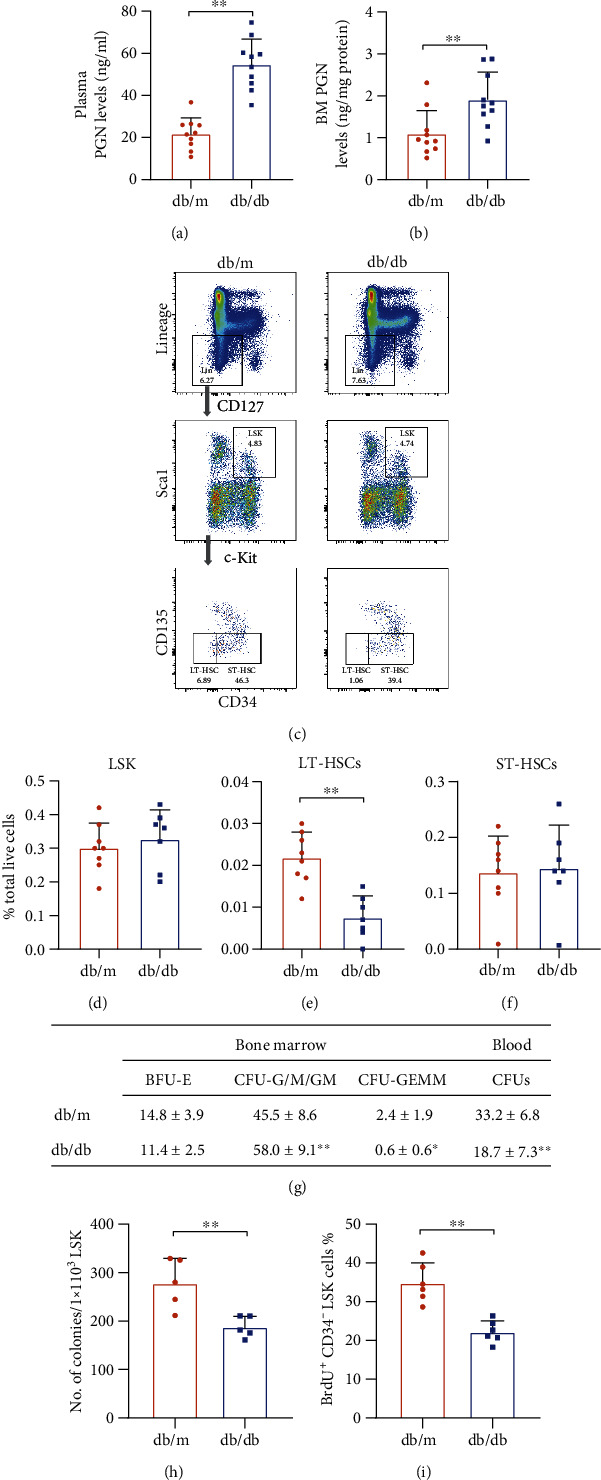
Diabetes increased the translocation of the bacteria-derived PAMP peptidoglycan and impaired HSPCs in the bone marrow. (a, b) ELISA was used to determine PGN levels in both the (a) plasma and (b) bone marrow extracellular fluid in diabetic db/db mice (*n* = 10 per group). (c) Representative gating scheme for the multicolor flow cytometry analysis of HSPC subpopulations. (d–f) The percentages of (d) LSK (Lin^−^CD127^−^Sca1^+^c-Kit^+^), (e) LT-HSC (Lin^−^CD127^−^Sca1^+^c-Kit^+^CD34^−^CD135^−^), and (f) ST-HSC (Lin^−^CD127^−^Sca1^+^c-Kit^+^CD34^+^CD135^−^) out of total live cells in the bone marrow from db/db mice that had diabetes for 4 months and the age-matched db/m control mice (*n* = 7 − 8 per group). (g) ACS-lysed cells from bone marrow or blood were plated in MethoCult GF M3434 medium for the CFU assay (*n* = 7 − 9 per group). (h) 1 × 10^3^ sorted LSK cells from db/m and db/db mice were cultured for 4 weeks and then plated in MethoCult GF M3434 in triplicate and tested for colonies in the LTC-IC assay (*n* = 5 per group). (i) The percentage of BrdU^+^CD34^−^LSK cells was analyzed by FACS after 2-day BrdU administration in BrdU incorporation assay (*n* = 6 per group). Data represent mean ± SD. PGN: peptidoglycan; BM: bone marrow; LT-HSC: long-term repopulating hematopoietic stem cell; ST-HSC: short-term repopulating hematopoietic stem cell. ^∗^*P* < 0.05, ^∗∗^*P* < 0.01.

Bacterial PAMPs can translocate from the intestine into the circulation and even to distant organs through impaired gut barriers in subjects with diabetes. We examined whether type 2 diabetes impacts the levels of a bacterial PAMP, PGN. We found that the PGN levels were markedly increased in db/db mice 4 months after the establishment of diabetes compared to their age-matched db/m controls (Figure 1(a)). We also observed increased PGN levels in extracellular fluid collected from db/db mouse bone marrow(Figure 1(b)), suggesting translocation of the bacterial antigen PGN to distant tissues. Bacterial antigens control the bone marrow stem cell pool. To assess whether increased PGN levels in the bone marrow of diabetic mice are associated with HSPC dysfunction, flow cytometry was used to identify the percentages of various HSPC subpopulations in the mouse cohort (Figure 1(c)). Even though the percentages of bone marrow Lin^−^/CD127^−^/Sca-1^+^c-Kit^+^(LSK) cells did not differ between the diabetic and control groups (Figure 1(d)), LK cells isolated from the diabetic bone marrow showed impaired migration ability in response to the chemoattractant CXCL12 and reduced proliferation compared to that of LK cells from db/m control mice (Suppl. Figure 1A and B). These parameters are key indicators of vasoreparative ability in vivo, as cells must self-expand and translocate from the bone marrow to the vessel injury site in response to chemoattractants. We next assessed the colony formation abilities of general HSPCs in both peripheral blood and bone marrow by the colony formation assay. After 10-day culture of ACS-lysed blood cells harvested from diabetic mice, those cells showed a marked decrease in total blood colony-forming units (CFUs). In the CFU assays of bone marrow cells, the total CFUs did not differ between db/m and db/db mice; however, the number of CFU-G/M/GM (CFU-granulocyte/monocyte/granulocyte, monocyte) was increased in diabetic mice compared to db/m mice, suggesting a shift in hematopoiesis towards proinflammatory cell types (Figure 1(g)). LSK cells were then subdivided into long-term (LT) and short-term (ST) repopulating HSCs; compared to those in the healthy controls, the number of LT-HSCs (Lin^−^/CD127^−^/Sca-1^+^c-Kit^+^/CD34^−^CD135^−^) was significantly reduced in the diabetic mice, while no change was observed in ST-HSCs (Lin^−^/CD127^−^/Sca-1^+^c-Kit^+^/CD34^+^CD135^−^) (Figures 1(e) and 1(f)). Because LT-HSCs represent the most primitive hematopoietic stem cells in the bone marrow, their reduced percentage supports that diabetes induces substantial hematopoietic deficiency in the bone marrow. We then performed the long-term culture initiating cells (LTC-IC) assay which is considered a surrogate in vitro assay for testing the function of the most primitive HSC population. db/db mouse LSK cells formed much less colonies than did db/m mouse LSK cells after the long-term culture (Figure 1(h)). Similar finding was seen in in vivo BrdU incorporation assay, as there was a 36% reduction of BrdU^+^ LT-HSCs in db/db mouse bone marrow (Figure 1(i)), suggesting diabetic LT-HSCs have impaired self-renewal capability. These data suggested that type 2 diabetes resulted in alteration of the bacterial antigen PGN in the circulation and bone marrow, which was accompanied by HSPC depletion and functional impairment.

### 3.2. Peptidoglycan Affects the Pool of Hematopoietic Stem/Progenitor Cells as well as Their Vasoreparative Functions in Mice with Diabetes

**Figure 2:**
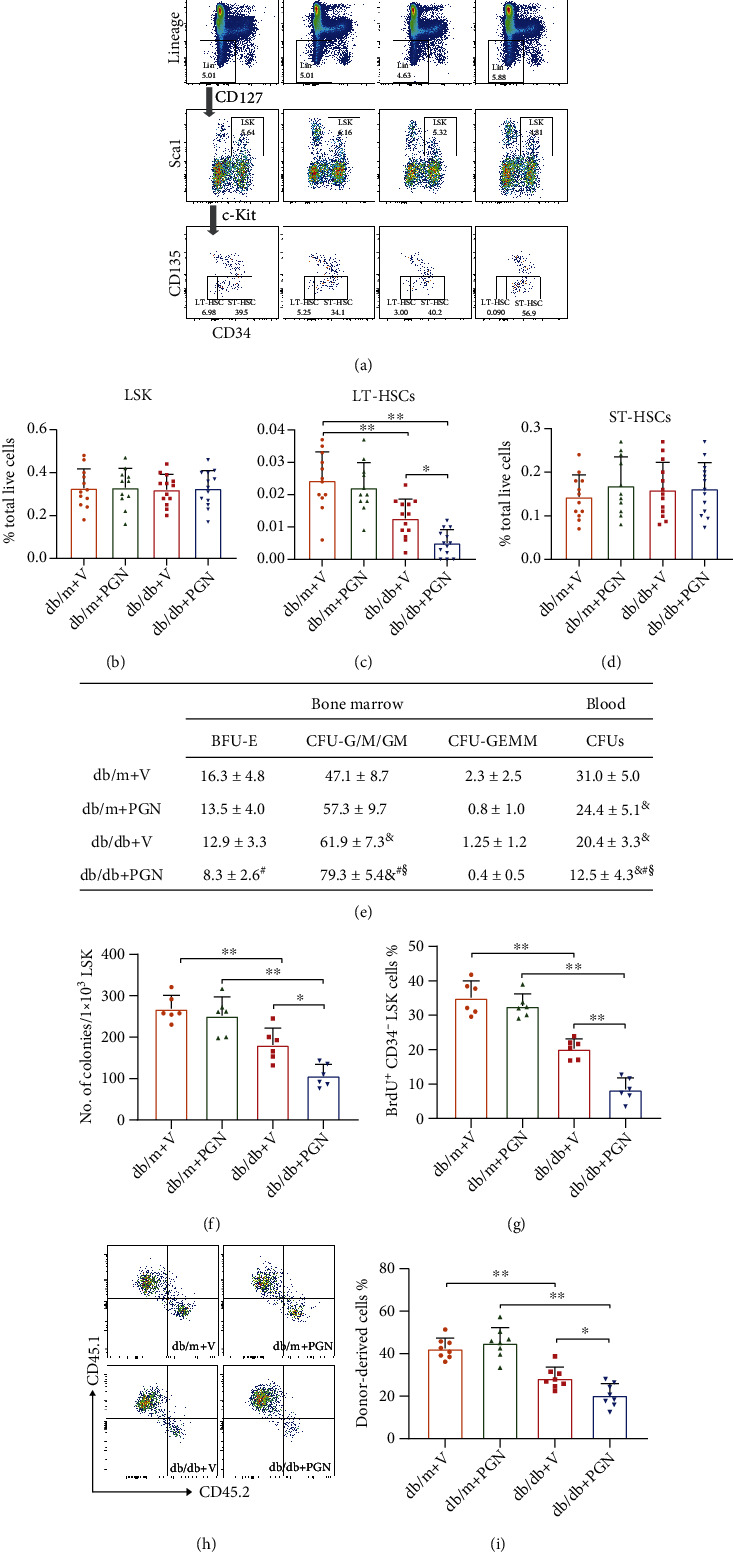
Peptidoglycan worsened diabetes-induced HSPC depletion and impairment in the bone marrow. (a) Representative flow cytometry gating scheme for the HSPC subpopulations in each group. (b–d) Flow cytometry analysis shows the percentage of (c) LT-HSC subpopulation was decreased in PGN-treated db/db mice group, while the percentages of (b) LSK and (c) ST-HSC were not affected by PGN injections (*n* = 11 − 13 per group). (e) Colony-forming capacity of bone marrow cells and blood cells from db/m and db/db mice treated with either PGN or vehicle control were tested in CFU assay. Colonies of BFU-E, CFU-G/M/GM, and CFU-GEMM using the bone marrow cells and total colony numbers using the peripheral blood were calculated after 10 days culture (*n* = 8 − 10 per group). (f) LSK cells from each group were grown in LTC-IC cultures for 4 weeks, followed by methylcellulose plating and culture for 12 days. PGN administration worsened diabetes-induced reduction in the total number of colonies per 1 × 10^3^ BM LSK cells. (g) The bar graph shows the percentage of BrdU^+^ CD34^−^LSK was the lowest in db/db+PGN mice by FACS in the in vivo BrdU incorporation study (*n* = 6 per group). (h, i) In the competitive transplantation study, lethally irradiated recipient mice (F1 progeny, CD45.1^+^ CD45.2^+^) were transplanted with 1 × 10^3^ LSK cells (CD45.2) from db/m+v, db/db+v, db/m+PGN, and db/db+PGN mice mixed with 3 × 10^5^ bone marrow cells (CD45.1, B6.SJL competitor). The bar graph in (i) shows the mean percentage of donor-derived cells in the peripheral blood 16 weeks after transplantation (*n* = 8 per group). Data represent mean ± SD. PGN: peptidoglycan; V: vehicle; LT-HSC: long-term repopulating hematopoietic stem cell; ST-HSC: short-term repopulating hematopoietic stem cell. ^∗^*P* < 0.05, ^∗∗^*P* < 0.01; ^&^compared to db/m+V; ^#^compared to db/m+PGN; ^§^compared to db/db+V.

To determine whether circulating PGN affected bone marrow HSPCs in mice with diabetes, db/db mice and their age-matched db/m controls were treated with 100 *μ*g of purified *E. coli* PGN every other day for 7 days via tail vein injection. As expected, increased circulatory levels of PGN caused a depletion of LT-HSCs in a type 2 diabetic mouse model without affecting the general LSK, ST-HSC, MPPs, CLPs, CMPs, GMPs, and MEPs percentages in the bone marrow, as well as circulating LSK population by flow cytometry analysis (Figures 2(a)–2(d) and Suppl. Figure 2A-D). Despite similar numbers of LSK cells observed in the 4 cohorts, PGN treatment exacerbated the diabetes-induced impairment of LK cell mobilization and proliferation functions (Suppl. Figure 2E-F). PGN injection worsened the diabetes-induced reduction in total blood CFUs, which also verified the effect of PGN on the differentiation ability of HSPCs. In addition, the number of CFU-G/M/GM in the bone marrow of db/db mice at 4 months of diabetes was increased as determined by the CFU assay. The number of CFU-G/M/GM of db/db mice treated with PGN was increased to a greater extent that than in mice treated with saline, suggesting that PGN worsened the diabetes-mediated hematopoietic shift towards a more proinflammatory cell type (Figure 2(e)). PGN treatment caused dramatic reduction in colonies forming by LTC-IC assay using LSK cells and in LT-HSC proliferation by in vivo BrdU corporation assay under diabetic condition, while no effect of PGN was observed in the control ones (Figures 2(f) and 2(g)). We further performed the competitive bone marrow transplantation assay to compare the bone marrow HSPC reconstitution potential among the 4 cohorts. Figures 2(h) and 2(i) show that db/db with PGN administration group had the lowest percentage of donor-derived cells compared to other groups.

### 3.3. Peptidoglycan Does Not Directly Impair the Migration and Proliferation Abilities of HSPCs from Diabetic Mice when Administrated Ex Vivo

**Figure 3:**
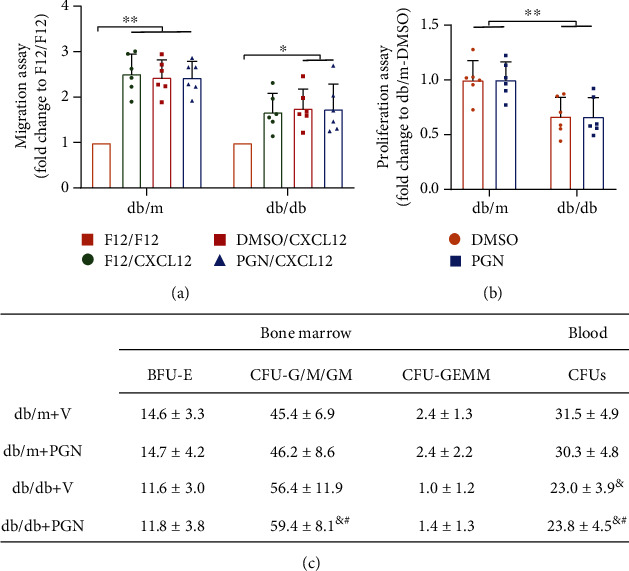
Ex vivo administration of PGN did not alter the vasoreparative functions or colony-forming ability of diabetic LK cells. (a, b) LK cells were isolated from db/m and db/db mice and treated with either PGN or vehicle ex vivo (*n* = 6 per group). (a) Ex vivo PGN treatment did not further blunt the migration of LK cells from diabetic mice to the chemoattractant CXCL12 by the fluorometric QCM 5 *μ*M96-well chemotaxis cell migration assay. (b) PGN-treated LK cells also showed similar proliferation ability measured by the proliferation BrdU ELISA kit in vitro. (c) CFU assay was used to determine the colony-forming abilities of BM and blood cells. The ex vivo administration of PGN did not alter the colony-forming abilities of BM and blood cells from diabetic mice (*n* = 8 − 10 per group). Data represent mean ± SD. PGN: peptidoglycan; V: vehicle. ^∗^*P* < 0.05, ^∗∗^*P* < 0.01; ^&^compared to db/m+V, ^#^compared to db/m+PGN.

To further explore the mechanisms underlying the deleterious effects of PGN on HSPC function, we isolated bone marrow LK cells from db/db mice with 4 months of diabetes and from their age-matched db/m controls and then treated them with either PGN or saline ex vivo. Surprisingly, PGN did not worsen the ability of LK cells from diabetic mice to migrate towards the chemoattractant CXCL12 compared to that of cells harvested from mice treated with saline (Figure 3(a)). Moreover, the proliferative abilities of LK cells harvested from diabetic mice treated with PGN and saline were similar (Figure 3(b)). We verified that the intravenous injection of PGN disrupted the hematopoietic balance in mice with diabetes by performing CFU assays; however, the colony-forming ability of LK cells from diabetic mice was unaffected by the ex vivo administration of PGN (Figure 3(c)). Collectively, these data indicate that PGN may aggravate diabetes-induced HSPC impairment via an indirect mechanism.

### 3.4. Peptidoglycan Affects the Sympathetic Nervous System in the Bone Marrow of Diabetic db/db Mice

**Figure 4:**
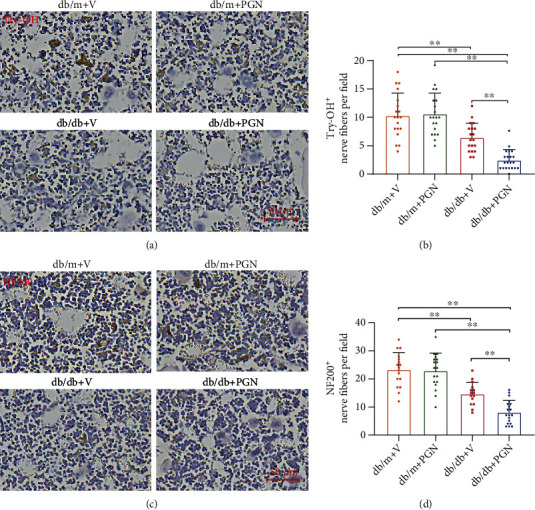
PGN administration dramatically decreased bone marrow innervation in diabetic db/db mice. (a) Representative images of immunohistochemical staining for tyrosine hydroxylase in femur cross-sections from mice in each group. (b) The number of Try-OH^+^ nerves was dramatically reduced in the bone marrow of db/db mice treated with PGN compared that in the bone marrow of mice treated with the vehicle control (*n* = 19 − 21 per group). (c) Representative images of immunohistochemical staining for NF200 in mouse femurs. (d) PGN further decreased the number of NF200^+^ nerve fibers in db/db mice (*n* = 15 − 19 per group). Data represent mean ± SD. PGN: peptidoglycan; V: vehicle; Try-OH: tyrosine hydroxylase; NF200: neurofilament 200. ^∗^*P* < 0.05, ^∗∗^*P* < 0.01.

Diabetes-mediated autonomic neuropathy has been reported to affect the vasoreparative functions of bone marrow stem cells. Recent studies indicate that bacterial antigens are prominently involved in the development of peripheral neuropathy. Thus, we hypothesize that increased PGN levels in subjects with diabetes results in bone marrow nerve disturbances, which ultimately lead to the impairment of bone marrow HSPCs. Consistent with previous findings, histological analysis of the femurs from db/db mice at 4 months after the establishment of diabetes revealed significant reductions in the numbers of tyrosine hydroxylase-positive (Try-OH^+^) nerves (Figures 4(a) and 4(b)). The intravenous administration of PGN further decreased the number of Try-OH^+^ nerve fibers in db/db mice (Figures 4(a) and 4(b)). As shown in Figures 4(c) and 4(d), intravenous injection of PGN also reduced the number of nerve endings by more than 40% in the bone marrow of diabetic mice, as determined by staining with neurofilament 200 (NF200). Taken together, these data demonstrate a deleterious effect of PGN on bone marrow sympathetic innervation.

### 3.5. A NOD1 Antagonist Restored the HSPC Pool Size and HSPC Function, which Was Associated with the Protection of Bone Marrow Autonomic Neurons

**Figure 5:**
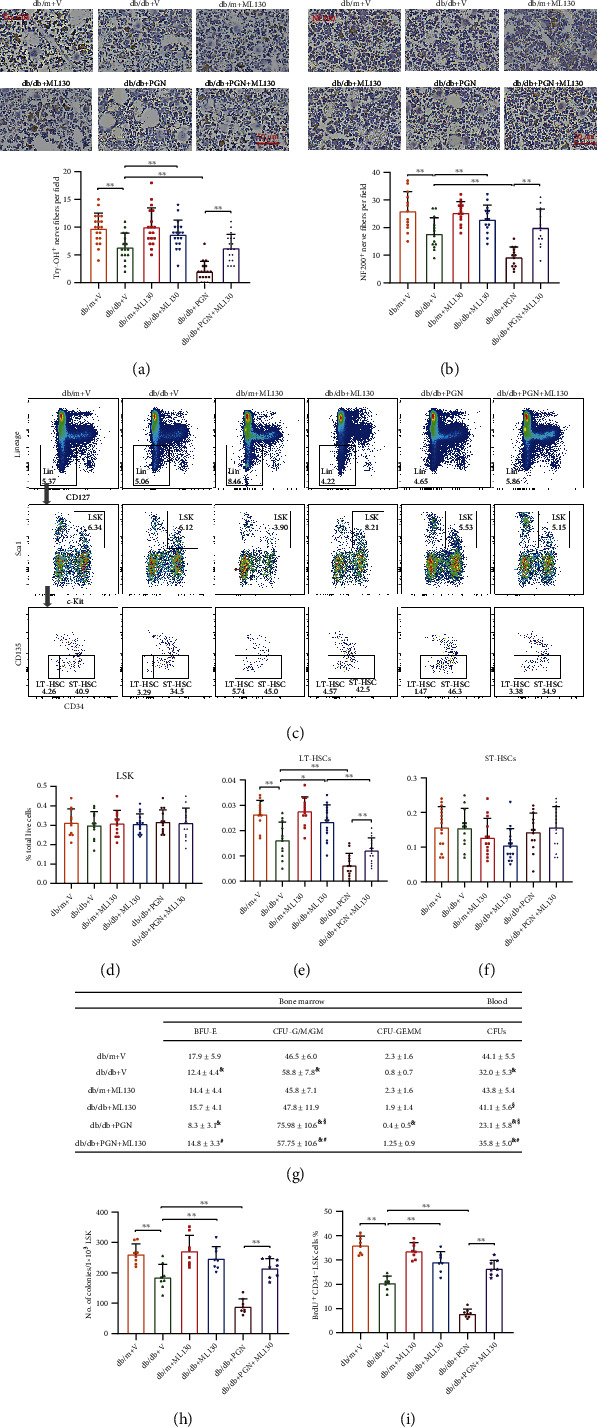
A NOD1-specific inhibitor ameliorated PGN-mediated neurodegeneration in the bone marrow of diabetic mice and ultimately restored HSPC numbers and functions. (a) The upper images show representative tyrosine hydroxylase IHC staining of the femurs of mice from each group. The bottom quantitative data show that ML130, a specific inhibitor of NOD1, restored the number of try-OH^+^ nerve fibers in the bone marrow of PGN-treated db/db mice (*n* = 16 − 19 per group). (b) The upper images show representative NF200 IHC staining of mouse femurs. The bottom quantitative data show that ML130 protected against the PGN-induced degeneration of NF200^+^ neurons in the bone marrow of db/db mice (*n* = 13 − 14 per group). (c) Representative images of HSPC subpopulations (LSK, LT-HSC, and ST-HSC) analyzed by flow cytometry. (d–f) ML-130 ameliorated the PGN-mediated depletion of bone marrow (e) LT-HSCs in diabetic mice. No changes in the percentages of the (d) LSK and (f) ST-HSC populations were observed among the groups (*n* = 11 − 15 per group). (g) CFU assay shows ML130 ameliorated the increase in the number of BM CFU-G/M/GM and the decrease in the number of total CFUs in the blood induced by PGN in diabetic mice (*n* = 12 per group). (h) 1 × 10^3^ LSK cells from each group were cultured and tested for colonies in the LTC-IC assay (*n* = 8 per group). (i) For the in vivo BrdU incorporation study, the percentages of LT-LSK cells in each group were analyzed by FACS (*n* = 8 per group). Data represent mean ± SD. PGN: peptidoglycan; V: vehicle; LSK: Lin^−^/CD127^−^/Sca-1^+^c-Kit^+^; LT-HSC: long-term repopulating hematopoietic stem cell; ST-HSC: short-term repopulating hematopoietic stem cell. ^∗^*P* < 0.05, ^∗∗^*P* < 0.01; ^&^compared to db/m+V; ^#^compared to db/db+PGN; ^§^compared to db/db+V.

We next investigated whether the inhibition of NOD1, a PGN receptor, could prevent the deleterious effects of PGN on bone marrow neuropathy and on the number and functions of HSPCs. ML130, a selective inhibitor of NOD1, was injected together with PGN into db/db or db/m mice every other day for 7 days. As shown in Figure 5(a), ML130 ameliorated the reduced number of Try-OH^+^ nerve fibers induced by PGN in mice with diabetes. The NOD1 inhibitor also protected against the deleterious effect of PGN on bone marrow autonomic neurons, as shown by NF200-positive staining (Figure 5(b)). In addition, blockade of NOD1 prevented the reduced percentages of LT-HSCs in db/db mice (Figures 5(c)–5(f)). ML130 restored PGN-induced migration and proliferation dysfunctions of general HSPC populations from diabetic bone marrow (Suppl. Figure 3A-B). ML130 also reestablished the hematopoietic balance that was disturbed by PGN in db/db mice as determined by CFU assays (Figure 5(g)). In the LTC-IC study, diabetic LSK cells from PGN treatment group showed very little colony forming after a long-term culture, while the number of colonies was markedly increased in those pretreated with ML130 suggesting ML130 is protective for maintaining the fitness of more primitive HSPCs (Figure 5(h)). ML130 also restored PGN-mediated reduction of BrdU^+^ LT-HSCs in db/db mouse bone marrow (Figure 5(i)).

### 3.6. Chemical Sympathectomy Mediated by 6-OHDA Blocks the Protective Effect of the NOD1 Antagonist on HSPCs

**Figure 6:**
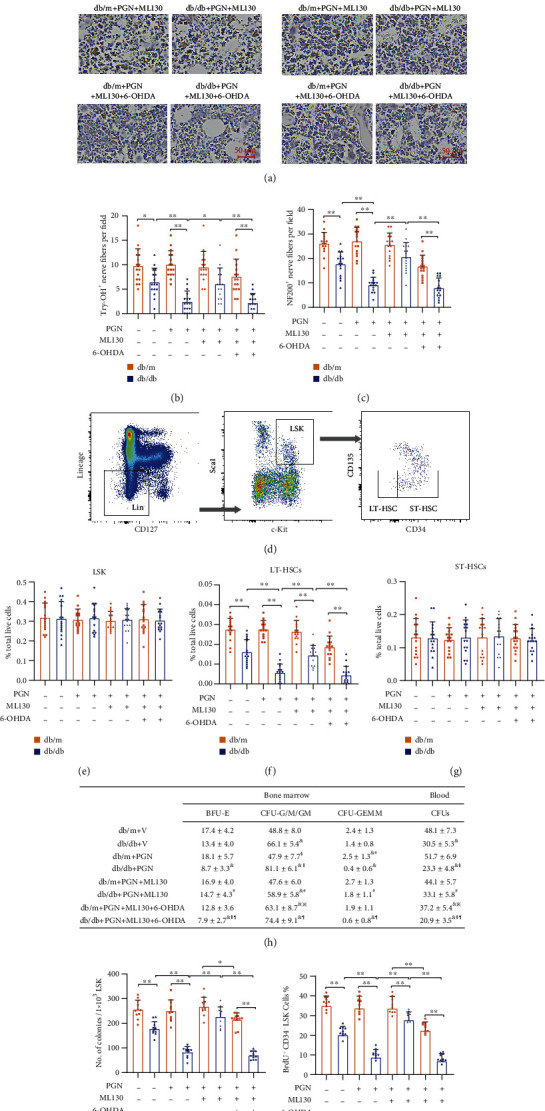
Chemical sympathectomy blocked the protective effects of ML130 on HSPC populations. (a) Representative IHC images of bone marrow sections stained with tyrosine hydroxylase (left panel) and NF200 (right panel). (b, c) db/db and db/m mice were treated with PGN, PGN+ML130, or vehicle after the chemical sympathectomy induced by 6-OHDA. 6-OHDA caused severe bone marrow denervation, as determined by the reduced numbers of (b) try-OH^+^ and (c) NF200^+^ neurons in the mouse femur (*n* = 16 per group). (d) Representative plots of flow cytometry analysis showing HSPC populations (LSK, LT-HSC, and ST-HSC). (e–g) The (f) LT-HSC percentage out of total live cells was dramatically reduced in sympathectomized (6-OHDA) diabetic animals regardless of ML130 treatment determined by multicolor flow cytometry studies. The (e) total LSK and (g) ST-HSC populations were not affected by 6-OHDA (*n* = 16 per group). (h) The CFU assay showed increased CFU-G/M/GM of bone marrow cells cotreated with 6-OHDA and ML130 compared to those treated with ML130 alone (*n* = 14 per group). (i) 1 × 10^3^ LSK cells from each group were cultured and tested for colonies in the LTC-IC assay. 6-OHDA blocked the beneficial effect of ML130 on PGN-induced reduction in the total number of colonies (*n* = 11 per group). (j) For the in vivo BrdU incorporation study, the bar graph in the (j) shows the percentages of BrdU^+^ CD34^−^LSK cells were decreased after he chemical sympathectomy induced by 6-OHDA irrespective of diabetic state by FACS analysis (*n* = 10 per group). Data represent mean ± SD. PGN: peptidoglycan; V: vehicle; LSK: Lin^−^/CD127^−^/Sca-1^+^c-Kit^+^; LT-HSC: long-term repopulating hematopoietic stem cell; ST-HSC: short-term repopulating hematopoietic stem cell. ^∗^*P* < 0.05, ^∗∗^*P* < 0.01; ^&^compared to db/db+PGN; ^§^compared to db/db+V; ^#^compared to db/db+PGN+ML130; ^※^compared to db/m+PGN+ML130; ^¶^compared to db/db+PGN+ML130.

To further confirm that the restorative effect of NOD1 inhibition on HSPCs occurred via the protection of bone marrow autonomic neurons, we evaluated the HSPC percentages and functions after chemical sympathectomy induced by 6-OHDA. As shown in Figures 6(a)–6(c), the bone marrow neuropathy in 6-OHDA-treated mice was severely reduced as determined by Tyr-OH and NF200 staining. As expected, 6-OHDA-induced sympathectomy blocked the protective effect of ML130 on the LT-HSC pool (Figures 6(d)–6(g)) as well as the migration and proliferation functions of general HSPC population (Suppl. Figure 3C-D). Chemical sympathectomy also disturbed the hematopoietic balance maintained by the NOD1 antagonist as determined by CFU assays (Figure 6(h)). Moreover, the LTC-IC study of LSK cells treated with the NOD1 inhibitor no longer showed improved numbers of colonies after chemical sympathectomy (Figure 6(i)). 6-OHDA also prevented the recovery of proliferation function of LT-HSCs mediated by ML130 treatment (Figure 6(j)). These data suggest that the PGN receptor affects HSPC subpopulations and functions via bone marrow autonomic neurons.

## 4. Discussion

This study reported for the first time that PGN is a key regulator of maintaining HSPC homeostasis in diabetes. Such gut-bone marrow cross talk adds a new dimension to the current understanding of the complex pathogenesis of microvascular complications in diabetes. This study further investigated the possible mechanisms by which PGN impairs HSPC function and uncovered the disturbance of sympathetic nervous system innervation in the bone marrow as a potent driver of imbalance of hematopoietic system. A selective inhibitor of NOD1 protected against PGN-induced autonomic neuropathy and therefore promotes bone marrow HSPC rejuvenation in diabetes.

Extensive evidence suggests that both the composition and function of the gut microbiome are altered in type 1 and type 2 diabetes [16–19]. Increased gut permeability in subjects with diabetes allows the translocation of bacteria, their metabolites, and PAMPs from the gut to distant organs, thereby causing systemic effects [20]. Diabetic nephropathy (DN) and diabetic retinopathy (DR) are two common microvascular complications of diabetes, and intestinal dysbiosis contributes to the development of DN and chronic kidney disease (CKD) [21]. A recent study showed that alteration of the microbiome resulted in elevated plasma acetate levels, thereby inducing the activation of the local renal renin-angiotensin system and the progression of early kidney injury in a diabetic rat model [22]. Our previous studies also suggest an association between the gut microbiome and retinal disorders in subjects with diabetes [5, 7]. The intermittent fasting prevented retinopathy by restructuring the gut microbiome and modulating microbe-derived metabolites in a diabetic db/db mouse model [7]. In another study [5], we compared the microbiome compositions and functions of diabetic Akita mice and their age-matched littermates using 16S rRNA sequencing and metatranscriptomic analysis and revealed that PGN biosynthesis pathways were remarkably activated in diabetic mice, thereby increasing the circulating PGN levels. We further determined that PGN directly acts on human retinal endothelial cells and impairs their adherent junctions in vitro [5].

PGN is a very large polymer consisting of glycan chains and amino acids. As a PAMP, PGN is an essential unit of the bacterial cell wall found in most Gram-positive and Gram-negative bacteria [23]. PGN has been reported to trigger chronic inflammation, thereby promoting diet-induced insulin resistance in adipocytes and hepatocytes [24, 25]. Here, we showed that increased circulating PGN lead to an elevated level of this type of PAMP in the local bone marrow of db/db mice, which selectively reduced the LT-HSCs and impaired reconstitution and proliferation abilities of primitive HSCs. We previously showed that PGN has a direct and detrimental effect on retinal microvascular endothelial cells through a Toll-like receptor 2-dependent pathway [5]. Since bone marrow-derived HSPCs are important for vascular health maintenance, impairment of their function has long been suggested to remarkably contribute to microvasculature injury in subjects with diabetes. Therefore, the novel finding of this study provides a possibility of PGN indirectly affecting diabetes microvascular complications by disturbing the bone marrow HSPC balance.

Interestingly, the deleterious effects of PGN on bone marrow innervation and LT-HSCs were only seen in diabetic mice, but not control ones, which suggests that there are some additional factors in diabetes that interact with the action of PGN. Our previous studies and others have shown that there were various protective mechanisms in healthy bone marrow which were compromised in diabetes, such as the activation of protective renin-angiotensin system (ACE2-Ang1-7-MAS axis), sufficient local levels of neuroprotective, and anti-inflammatory factors, as well as normal peripheral circadian rhythm [5, 26–29]. Therefore, it is possible that when those beneficial factors were perturbed by diabetes, they were unable to counteract the deleterious effects of PGN. Extensive investigation to understand the precise regulatory complex network of the bone marrow homeostasis is required.

The concept of the gut-bone marrow axis is supported by evidence that the gut microbiome is an extrinsic modulator of the hematopoietic system. In the clinic, bone marrow suppression is often observed in patients administered antibiotics for prolonged periods [30]. In animal studies, mice receiving antibiotics also exhibited impaired hematopoiesis with marked suppression of HSPC populations due to the depletion of intestinal flora [31]. Alteration of the microbiome composition in a *Rag*^−/−^ mouse model led to marked reductions in the LSK, LT-HSC, and ST-HSC populations, and the transplantation of feces from wild-type mice reversed such changes in the bone marrow of knockout mice [32]. The complexity of the gut microbiome is also strongly correlated with HSPC functions. Cytokine-induced HSPC mobilization was reduced in antibiotic-treated mice, suggesting that endotoxins, as potent cofactors, are involved in HSPC trafficking in the bone marrow [33].

Bioactive secondary metabolites of bacteria and PAMPs are considered major messengers in cross talk between the microbiome and bone marrow. Lipopolysaccharide (LPS) is one of the most well-studied PAMPs in diabetes and has been found to disturb the hematopoietic balance [34, 35]. LPS administration drives quiescent LT-HSCs into the cell cycle and promotes the differentiation of HSPCs towards a more proinflammatory cell type, possibly via the direct activation of Toll-like receptor 4 on the cell surface [36, 37]. On the other hand, LPS acts on nonhematopoietic cells, such as endothelial cells and mesenchymal stromal cells, and promotes the secretion of proinflammatory cytokines in the hematopoietic microenvironment, which indirectly affects HSPCs by engineering the bone marrow niche [38–41]. However, the effects of other PAMPs on HSPC populations, including PGN, have remained elusive, especially in the context of diabetes.

We previously performed genomic and metatranscriptomic analysis of the microbiome and revealed that PGN biosynthesis pathways were remarkably enriched, which resulted in increased circulatory levels of the PAMP in subjects with both type 1 and type 2 diabetes. However, whether increased circulating PGN levels modulate bone marrow cells in diabetes remains largely unknown. PGN reportedly translocates from the gut to the bone marrow and promotes neutrophil functions via a NOD1-dependent pathway [42]. Another study showed that PGN accelerates granulopoiesis by TLR2-dependent paracrine mechanisms and participates in innate immunity and host defense [10]. In our current study, we report for the first time that PGN administered in vivo not only modulated the HSPC pool size but also regulated their migration, proliferation, and reconstitution in subjects with diabetes. PGN receptors are widely expressed on hematopoietic cells, including HSPCs [43]. Interestingly, unlike PGN administration via the tail vein, the ex vivo administration of PGN did not worsen HSPC migration and proliferation, suggesting that the effects of PGN on HSPCs in subjects with diabetes are not attributable to the direct binding of cell surface receptors.

The bone marrow in subjects with diabetes is characterized by neuropathy, microangiopathy, and inflammation [2]. As a result, alteration of the bone marrow microenvironment disrupts the hematopoietic system, causing imbalance and functional impairment of HSPCs, which finely regulates the process of myelopoiesis and the pool size of progenitor cells with vasoreparative potential [2, 11]. From a clinical perspective, the depletion of HSPCs is associated with the progression of microvascular complications in patients with diabetes and is a predictor of adverse outcomes [28, 44]. PGN receptors were reported to be highly expressed in neurons, and a transgenic mouse model of PGN-sensing molecules showed altered expression levels of synaptic-related genes and behavioral disorders, suggesting a possible association between PGN and the central neuron system [45, 46]. Direct PGN injection into the mouse brain parenchyma induced microglial cell activation and neurotoxicity via the PI3K-dependent signaling pathway [47]. We investigated whether the in vivo injection of PGN could impair HSPC function by compromising bone marrow autonomic neuropathy, as we did not observe a direct effect of PGN on HSPCs ex vivo. In this study, we found that the PGN levels were elevated in not only the circulation but also the local bone marrow. PGN administration exacerbated the diabetes-induced reductions in both NF200^+^ and Try-OH^+^ sympathetic nervous system fibers, which was associated with a reduction in HSPC numbers and impairment of cell functions. To further verify our hypothesis, 6-OHDA was used to induce chemical sympathectomy. The LT-HSCs in mice treated with 6-OHDA were severely depleted, and their vasoreparative functions and reconstitution function were compromised; these deficiencies could not be corrected by specially inhibiting the PGN receptor NOD1. This novel finding innovatively demonstrates that the microbiota is associated with peripheral neuropathy and hematology in the context of diabetes and furthers our understanding of the precise mechanisms underlying diabetic bone marrow neuropathy and HSPC modulation.

Another novel finding of the study is that PGN affects bone marrow HSPCs via a NOD1-dependent pathway. Multiple proteins sense PGN and its fragments, including NOD1, TLR2, PGN recognition protein 1, and NOD-, LRR-, and pyrin domain-containing 3 [23]. We previously performed metatranscriptomic analysis and revealed that the enriched PGN biosynthesis pathways in the microbiota of diabetic mice were mainly responsible for the production of meso-diaminopimelic (meso-DAP) acid-containing muropeptides, which are specifically recognized by the NOD1 receptor [5]. NOD1 activation participates in multiple biological processes, such as innate immune regulation, chronic inflammation, and insulin resistance [25]. Consistent with our previous RNA sequencing data, our current study showed that a specific NOD1 inhibitor prevented PGN-induced bone marrow neuropathy and HSPC deprivation, further confirming the critical role of NOD1 in PGN-mediated bone marrow defects in subjects with diabetes.

## 5. Conclusions

In summary, our study suggests for the first time the essential role of a microbial PAMP, PGN, in diabetes-induced HSPC depletion and impairment of their vasoreparative and reconstitution functions and highlights the importance of NOD1-dependent autonomic neuropathy in this gut and bone marrow communication. These findings have expanded our understanding of the mechanisms underlying diabetes-mediated bone marrow pathology and provide a novel therapeutic option for bone marrow rejuvenation for the prevention and treatment of diabetic vascular complications.

## Data Availability

All data generated or analyzed during this study are included in this published article.

## Supplementary Materials

Supplementary Materials Figure S1: diabetic HSPCs in the bone marrow showed impaired migration and proliferation function. Figure S2: the percentages of hematopoietic lineages and blood LSK cell measurements and HSPC function evaluation in PGN-treated diabetic mice. Figure S3: ML130 ameliorated PGN-induced dysfunctions of bone marrow LK cells which was blocked by chemical sympathectomy.
