# Supplementary material for: Peptidoglycan-Mediated Bone Marrow Autonomic Neuropathy Impairs Hematopoietic Stem/Progenitor Cells via a NOD1-Dependent Pathway in db/db Mice
Source: Stem Cells Int. 2022 Aug 4;2022:4249843. doi: 10.1155/2022/4249843 (PMC9371813; doi:10.1155/2022/4249843)
Supplement: Supplementary Materials — Figure S1: diabetic HSPCs in the bone marrow showed impaired migration and proliferation function. Figure S2: the percentages of hematopoietic lineages and blood LSK cell measurements and HSPC function evaluation in PGN-treated diabetic mice. Figure S3: ML130 ameliorated PGN-induced dysfunctions of bone marrow LK cells which was blocked by chemical sympathectomy. [file 4249843.f1.docx]

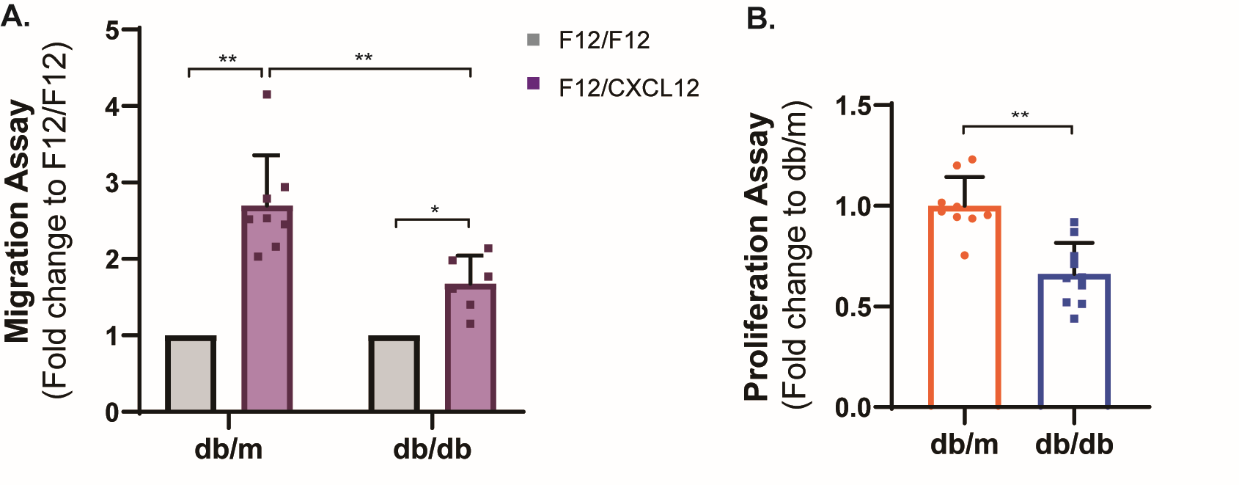


**Supplemental Figure 1. Diabetic HSPCs in the bone marrow showed impaired migration and proliferation function.** A: Migration function of LK cells from diabetic and control mice bone marrow was measured by testing their migration ability toward chemoattractant CXCL12 using the fluorimetric QCM 5 μM 96-well chemotaxis cell migration assay (n = 6-8 per group). B: BrdU ELISA kit was used to measure the proliferation ability of bone marrow LK cells from db/db mice at 4 months of diabetes and their age matched controls (n = 9-10 per group). Data represent mean±SD. **P<0.01.


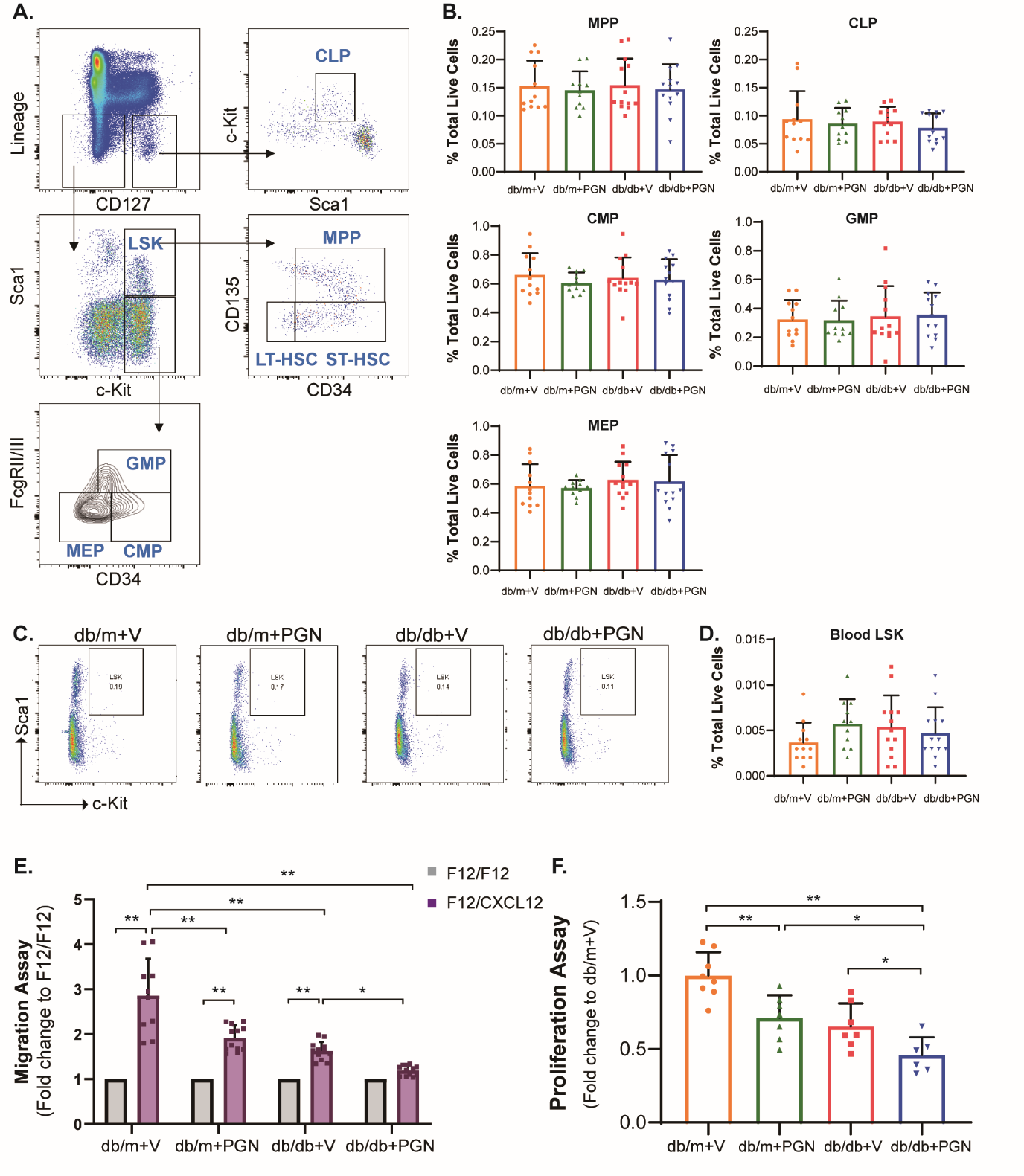


**Supplemental Figure 2. The percentages of hematopoietic lineages and blood LSK cells measurements and HSPC function evaluation in PGN treated diabetic mice.** A: The gating scheme of hematopoietic lineages for flow cytometry studies enumerating bone marrow MPPs, CLPs, CMPs, GMPs and MEPs percentages. B: No obvious changes were found in the percentages of MPPs (Lin^-^CD127^-^Sca1^+^c-Kit^+^CD34^+^CD135^+^), CLPs (Lin^-^CD127^+^Sca1^med^c-Kit^med^), CMPs (Lin^-^CD127^-^Sca1^-^c-Kit^+^CD34^+^FcgRII/III^lo^), GMPs (Lin^-^CD127^-^Sca1^-^c-Kit^+^CD34^+^FcgRII/III^hi^) and MEPs (Lin^-^CD127^-^Sca1^-^c-Kit^+^CD34^-^FcgRII/III^lo^) by FACS among the four groups (n= 11-13). C: The gating plots are representative images of the LSK cells in the peripheral blood in the four cohorts. D: The percentage of blood LSK cells in db/db+PGN group was similar to those in db/db treated with vehicle (n= 11-13). E and F: LK cells from the bone marrow of db/db mice treated with PGN showed impaired migration function measured by chemotaxis cell migration chamber (E) and proliferation (F) abilities using BrdU ELISA kit (n = 6-12 per group). Data represent mean±SD. **P*<0.05, **P<0.01.


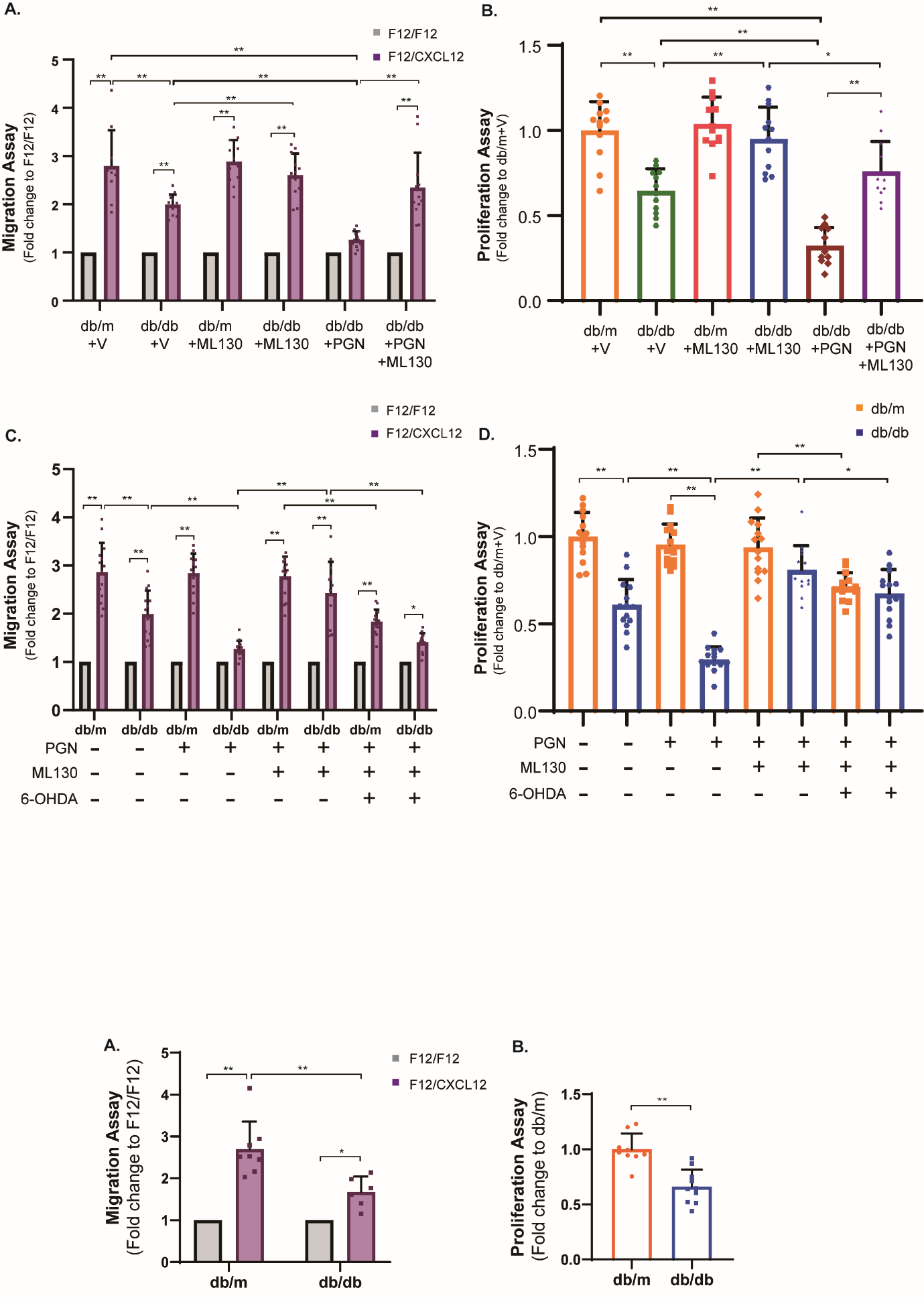


**Supplemental Figure 3. ML130 ameliorated PGN-induced dysfunctions of bone marrow LK cells which was blocked by chemical sympathectomy.** A and B: The NOD1 inhibitor (ML130) restored the migration (A) (n = 10-12 per group) and proliferation (B) (n = 12 per group) vasoreparative functions of HSPCs from the bone marrow of diabetic mice treated with PGN. C: ML130 failed to promote the migration of HSPCs towards the chemoattractant CXCL12 in diabetic mice cotreated with 6-OHDA using chemotaxis cell migration chamber (n = 14 per group). D: 6-OHDA blocked the protective effect of ML130 on HSPC proliferation by BrdU ELISA kit (n = 14 per group). Data represent mean±SD. **P*<0.05, **P<0.01.
